# Cloud Based Metalearning System for Predictive Modeling of Biomedical Data

**DOI:** 10.1155/2014/859279

**Published:** 2014-04-14

**Authors:** Milan Vukićević, Sandro Radovanović, Miloš Milovanović, Miroslav Minović

**Affiliations:** Faculty of Organizational Sciences, University of Belgrade, Jove Ilića 154, 11000 Belgrade, Serbia

## Abstract

Rapid growth and storage of biomedical data enabled many opportunities for predictive modeling and improvement of healthcare processes. On the other side analysis of such large amounts of data is a difficult and computationally intensive task for most existing data mining algorithms. This problem is addressed by proposing a cloud based system that integrates metalearning framework for ranking and selection of best predictive algorithms for data at hand and open source big data technologies for analysis of biomedical data.

## 1. Introduction

Data mining can be defined as the process of finding previously unknown patterns and trends in databases and using that information to build predictive models [1]. Due to increasing amount of data generated in healthcare systems (medical records, gene expression data, medical image data, etc.), analysis became too complex and voluminous for traditional methods and this is why data mining is becoming increasingly important [2].

In the last decade data mining techniques (like clustering, classification, or association) were successfully applied on different medical and biomedical problems like prediction of heart attacks [3], diagnostics based on gene expression microarray data [4], classification of Parkinson's disease [5], identification of liver cancer signature [6], and so forth.

Special area of medical data mining is biomedical data mining that seeks to connect phenotypic data to biomarker profiles and therapeutic treatments, with the goal of creating predictive models of disease detection, progression, and therapeutic response. This area includes mining genomic data (and data from other high-throughput technologies such as DNA sequencing and RNA expression), text mining of the biological literature, medical records, and so forth, and image mining across a number of modalities, including X-rays, functional MRI, and new types of scanning microscopes [7].

**Figure 1:**
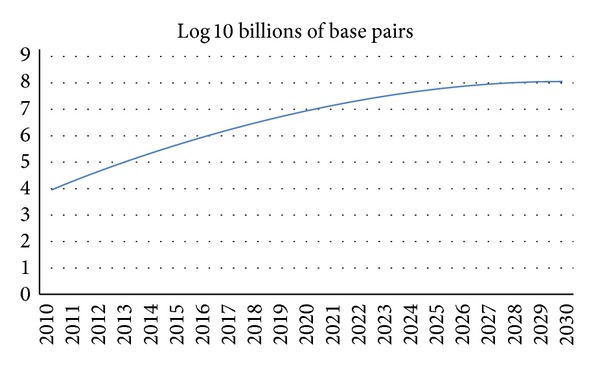
Projected growth of DNA sequence data in the 21st century [7].

Even though many algorithms were specially designed for application in this area [8], the exponential increase of genomic data brought by the advent of the third generation sequencing (NGS) technologies and the dramatic drop in sequencing cost have posed many challenges in terms of data transfer, storage, computation, and analysis of big biomedical data [2, 7, 9, 10]. These authors emphasize the lack of computing power and storage space, as a major hurdle in achieving research goals. They propose cloud computing as a service model sharing a pool of configurable resources, which is a suitable workbench to address these challenges (Figure 1).

One of the “soft” approaches for reducing the need for computer power for data analysis is introduction of metalearning systems for selection and ranking of the best suited algorithms for different problems (datasets). These systems store historical experimental records (descriptions of datasets and algorithm performances) and, based on these records, evolve models for prediction of algorithm performances on a new dataset. By using these systems, analyst does not have to evaluate large number of algorithms on a big data (only ones with the best predicted performance) and this way saves computational and time resources. Even though specialized metalearning systems are developed for many application areas like electricity load forecasting [11], gold market forecasting [12], choosing metaheuristic optimization algorithm for traveling salesman problem [13], and so forth, there are not many researches that utilize metalearning in medicine [14] and in this paper we will propose such approach. Similar efforts have been made in the field of continuous improvement of business performance with big data [15], which lets users analyze business performance in distributed environments with a short response time, which can be analogous with biomedical systems.

Researchers in this area suggest that the main problem of exponential data growth is to provide adequate computing infrastructure that has the possibility to assemble, manage, and mine the enormous and rapidly growing data [2, 7, 9, 10]. They emphasize that intersection point between genome technology, cloud computing, and biological data mining provides a launch pad for developing a globally applicable cloud computing platform capable of supporting a new paradigm of data intensive, cloud-enabled predictive medicine.

In this paper we propose an extension of cloud based systems [16, 17] with data and model driven services based on metalearning approach. Additionally, this system includes open source data mining environments as a platform service for users. System is based on open source technologies and this is very important since they enable collaborative collection of data and fast development of new algorithms [18].

## 2. State of the Art

In this section a brief overview of cloud based healthcare systems and metalearning systems which are correlated with proposed system is discussed. Cloud systems emerged as a technology breakthrough in the last decade and impacted a wide range of business like SME [19], education [20], e-government [21], data mining [22], and so forth.

Ahuja et al. [23] exhaustively reviewed usage and consideration points in implementing cloud healthcare system. They identified that the most important points are infrastructure and number of facilities. Infrastructure has great influence since most of the healthcare facilities and office locations were built years ago and cannot use cloud systems. Number of facilities is important on operation of health organization and whether their IT infrastructure is distributed between facilities or is in a single datacenter. Moving to the cloud would help communication, application, and collaboration between health organizations. Cloud computing reduces operating costs, because the need for IT staff in each facility is lower and overall IT budget is reduced.

The advantages of cloud computing and big data technologies, like Hadoop and related software, increased their popularity in medicine and bioinformatics. Dai et al. [16] identified four bioinformatics cloud services. Those are DaaS (data as a service), SaaS (software as a service), PaaS (platform as a service), and IaaS (infrastructure as a service). Bioinformatics generates huge amount of raw data and they should be available for data analysis through DaaS. Additionally, a large diversity of software tools is necessary for data analysis and SaaS is provided as an option in regarding this problem. Platform as a service provides programmable platform for development, testing, and deploying solutions online. IaaS offers a complete computer infrastructure for bioinformatics analysis.

Lack of support for complex and large scale healthcare application of electronic medical records was identified by Li et al. [24]. Therefore, XML was used as a model for managing medical data while Hadoop infrastructure and MapReduce framework were used for data analysis. Their system, called XBase, is doing various data mining tasks like classification of heart valvular disease, detecting association rules, diagnosis assistance, and treatment recommendation.

As Schatz et al. [25] stated, sequencing of DNA chain is improving at a rate of about 5-fold per year, while computer performance is doubling only every 18 or 24 months. Therefore, addressing the issue of designing data analysis arises as a question. A practical solution for solving this problem is to concentrate on developing methods that make better use of multiple computers and processors, where cloud computing emerges with promising results. They stated that Hadoop/MapReduce technology is particularly well suited, from genomic point of view, for analysis of DNA sequence. The Crossbow genotyping program leverages Hadoop/MapReduce to launch many copies of the short read in parallel leveraging of Hadoop/MapReduce and Crossbow for greater results. In their benchmark test on the Amazon cloud, Crossbow Hadoop/MapReduce analyzed 2.7 billion data points in about 4 hours, which included the time required for uploading the raw data, for a total cost of $85 USD. Beside this, they described obstacles which can pose significant barrier in analysis of DNA sequence.

An interesting approach to design of biomedical cloud system was described by Taverna [26]. It is a workflow management system that allows uploading data to the cloud from web application, creating data flow and run analysis from multiple computing units. While analysis is running, user can monitor the progress. This application also allows sharing of data flow, enabling many researchers from the same or other projects to influence and give contribution to research process.

Frameworks for cloud based genome data analysis, such as Galaxy [27], offer generalized tools and libraries as components in workflow editor. Galaxy enables users to define pipelines that through specifically developed visualization show progression of the workflow. It is extensible and, therefore, a community built around it contributes in developing various tools for genome analysis. It is important to notice that Galaxy is integrated with biomedical databases, such as UCSC table data, BioMart Central, and modENCODE server.

Hadoop and MapReduce distributed computing paradigm has been implemented in CloudBurst [28]. It is used for mapping short reads to reference genomes in a parallel fashion in cloud environment. Essentially, it provides a parallel read-mapping algorithm optimized for mapping sequence data to the human genome and other reference genomes, intended for use in a biological analysis including SNP discovery, genotyping, and personal genomics.

Another large biological extensible workbench is SeqWire [29]. Users are allowed to write and share pipeline modules. It provides massive parallel processing using sequencing technologies, such as ABI SOLID and Illumina, web application, pipeline for processing and annotating sequenced data, query engine, and a MetaDB.

Web based cloud system, such as FX [30], provides high usability for users that are not familiar with programming techniques. It is developed for various biomedical data analyses such as estimating gene expression level and genomic variant calling from the RNA sequence using transcriptome-based references. User uploads data and configures data analysis settings on Amazon Web Service (AWS). Since this application is domain specific, it does not require manual arrangement of pipelines.

Critical cloud services in biomedicine are infrastructure services. Therefore, CloVR [31] offers a virtual operating system with preinstalled packages and libraries required for biomedical data analysis, such as large-scale BLAST searches, whole-genome assembly, gene finding, and RNA sequence analysis. It is implemented as an online application but does not provide GUI. Instead, command-line based automated analysis pipelines with preconfigured software packages for composing workflows are implemented.

Chae et al. [9] focus on two emerging problems in bioinformatics data analysis. Those are computation power and big data analysis for the biomedical data. Biomedical analysis requires very big computing power with huge storage space. They proposed BioVLab as an affordable infrastructure on the cloud, with a graphical workflow creator which provides an efficient way to deal with these problems. BioVLab consists of three layers. The first layer is a graphical workflow engine, called XBaya, which enables the composition and management of scientific workflows on a desktop. The second layer, gateway, is a web-based analysis tool for the integrated analysis of microRNA and mRNA expression data. Analysis is done on Amazon S3 Interface, which presents third layer of architecture. Data and commands from gateway are transferred to cloud, which analyze data and return results to user on desktop. They emphasized that analysis of big medical data requires use of appropriate tools and databases from a vast number of tools and databases; therefore using cloud would not solve problems of computational power and big data analysis.

Cloud healthcare application could have great impact on society, but security, privacy, and government regulation issues limit its usage. Zhang and Liu [32] defined security model for cloud healthcare system. First part of the model is secure collection of data. Data collection module is created and maintained independently by care delivery organizations. Second part is secure storage. Storage system must be encrypted and it can allow only authorized access. Third part is secure usage, which consists of medicine staff signature and verification sections. Their model deals with problem of information ownership, authenticity and authentication, nonrepudiation, patient consent and authorization, integrity and confidentiality of data, and availability of system.

Since security is identified as a major challenge in cloud systems, Wooten et al. [33] designed and implemented secure healthcare cloud system. This was achieved with a trust-aware role-based access control and a tag system. System was implemented on top of Amazon Web Services (AWS) Elastic Compute Cloud (EC2) and Linux, Apache, MySQL, and PHP (LAMP) solution stack.

Liu and Park [34] focused on challenges and adaptation of e-healthcare cloud systems. This system extends the cloud paradigm in order to satisfy global demands in digital healthcare applications. Therefore, technology, healthcare process, and service are identified as the main characteristics of healthcare cloud systems. Similarly, new challenges arose by the unique requirements of the e-healthcare industry for using cloud services for regulation, security issues, access, intercloud connectivity, and resource distribution.

IBM Watson is also used in healthcare as cloud service. Giles and Wilcox [35] used Watson ability to use natural language processing and combine it with content analysis in order to help medical staff in diagnostic analysis. This application of Watson identifies diseases, symptoms, right medications, and modifiers directly from medical records from different medical facilities stored on cloud.

Knowledge cloud based systems in medicine, as Lai et al. [17] stated, are one of the major government's strategic plans to drive the healthcare services which are identified as public concerns in China. They highlighted some successful criteria for establishment of a private knowledge network for business network collaboration and the knowledge cloud system for radiotherapy dynamic treatment service in China, such as innovation outsourcing, marketing opportunity, economy of scales, leverage existing resources, and service on demand. Three parties are identified in the KaaS service model. The first party is the* knowledge user* (patients, hospitals, and doctors), the one who pays for the knowledge service on demand. The second party is the* knowledge expert* (external consultants), the one who provides the knowledge service on demand. The third party is the* knowledge agent* who links together the knowledge user and the knowledge expert on demand.

Great potential of cloud services in the area of biomedicine is identified by Grossman and White [7], who made a vision of biomedical cloud in the future. Since amount of data which hospitals and medical institutions are dealing with is growing rapidly, big data technologies will have indispensable role in data analysis. Consequently, managing and processing data will fundamentally change, and new data mining and machine learning algorithms will be developed to deal with these changes. Explosion of data is expected to be in genomic, proteomic, and other “omic” data and molecular and system biology. Probes that collect data from tissue are more complex and allow simultaneous tracking and collect more data than few years ago. Authors have also considered several issues such as security, scalability of storage, scalability of analysis, peer with other private clouds, and peer with public clouds.

Metalearning presents powerful methodology which enables learning on its past knowledge of solving different tasks. This methodology is used in fraud detection [36], time series forecasting [37], load forecasting [11], and others.

## 3. Metalearning Framework for Clustering Biomedical Data

Exponential growth of the data and rapid development of large number of complex and computationally intensive data mining algorithms led to one of the major problems in modern data mining: selection of the best algorithm for data at hand [38]. Namely, analyst often does not have enough time or resources for creating models and evaluating them with all available algorithms.

One of the most promising approaches for dealing with this problem is metalearning [39, 40]. Metalearning is methodology which solves different data mining tasks based on past knowledge. The main idea is to store history of experimental results with descriptions (meta-attributes) of datasets (e.g., dataset characteristics, algorithm, and classification accuracies for classification problems) and, based on this, to create metamodel (classification or regression) that will predict the performances of each algorithm on new dataset. Creating of such metamodel (with good performance) would reduce the need for brute force evaluation (evaluation of every algorithm).

Metalearning system is built on set of algorithm or combination of algorithms (ensembles). Therefore, every algorithm or combination of algorithms (ensembles) is simpler. Theoretically, metalearning system can be infinitely large by putting metalearning as component of other metalearning systems. An advantage of its using is that it can address new types of tasks that have not been seen but are similar to already defined problems.

Metalearning, by Smith-Miles [39, 40], is defined with the following aspects:

(i) the problem space, *P*, which represents set of instances (datasets) of a given problem class;
(ii) the meta-attribute space, *M*, which contains characteristics that describe existing problems (e.g., number of attributes, entropy, normality, etc.);
(iii) the algorithm space, *A*, which represents the set of candidate algorithms which can be used to solve the problems defined in problem space *P*;
(iv) a performance metric, *Y*, which represents measures of performance of an algorithm on a problem (e.g., classification accuracy (for classification problems) or root mean square error (for regression problems)).

General procedure for metalearning is done in several steps: first, datasets from problem space are evaluated by algorithms from algorithm space. Further, metafeatures of the datasets are related to algorithm performance, forming the database of metaexamples. Then, regression or classification models are created (and evaluated by performance metric). Finally, when new problem (dataset) arrives, meta-attributes are extracted and performance prediction is made. In this way analyst does not have to evaluate each algorithm on each dataset but only ones with the best predicted performance of the problem and algorithm spaces. While technologies for data collection enabled cheap and fast accumulation of data and extension of problem space, development and collection of data mining algorithms is a more difficult problem since development of new algorithms demands a lot of time and effort, and also different algorithms are implemented on different platforms.

The most important issue for good performance of metalearning systems is the size of problem and algorithm space because the accuracy of metamodels is directly dependant on these spaces [39, 40]. This means that cloud based system and service oriented architecture should be natural environment for this kind of systems because it would enable community based extension models to be created and evaluated by performance metric in order to capture relations between meta-attributes and algorithm performance.

With metalearning approach in solving problems time for choosing appropriate algorithm for problem is greatly reduced but requires time for creating and updating metamodels, especially if data and algorithms are gathered from community. This is one of the main motivations for integration of such a system in cloud based environment and integration with big data technologies (like Hadoop, Hive, and Mahout) for storing and aggregation of data and predictive modeling.

### 3.1. Component Based Metalearning System for Biomedical Data

One of the promising approaches for tackling these problems (existence of large algorithm space and existence of efficient procedure for selection of the best algorithm) is component based data mining algorithm design [41–43]. This approach divides algorithms with similar structure (in this case representative based algorithms) into parts with the same functionality called subproblems. Every subproblem has standardized I/O structure and can be solved with one or more reusable components (RCs), presented in Table 1. This approach combination of RCs, which originates from different algorithms, can be used to design large number (thousands) of new “hybrid” algorithms. This approach gave very promising results in the area of clustering biomedical (gene expression) data [8, 44, 45].

Combining RCs is used for reproducing or creation of cluster algorithms. For example,* K*-means algorithms can be reconstructed as RANDOM-EUCLIDEAN-MEAN-COMPACT. However, a new hybrid algorithm can be constructed using DIANA-CORREL-MEDIAN-CONN, where DIANA is used to initialize representatives, CORREL to measure distance, MEDIAN to update representatives, and CONN to evaluate clusters.

**Figure 2:**
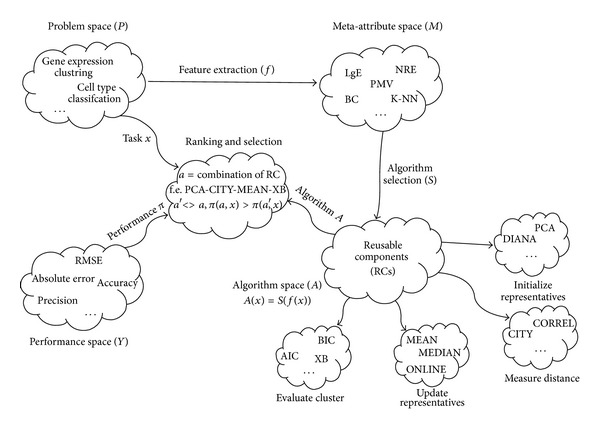
Extended metalearning system for clustering biomedical data.

Extended metalearning system, shown in Figure 2, is used in this research. Problem space is presented in upper left cloud. Every problem (dataset) from problem space *P* has its task (clustering, classification, regression, etc.) denounced *x*. Based on problem, function *f* extracts meta-attributes. For selected problem, based on meta-attributes, function *S* selects algorithm from algorithm space *A*. Every algorithm is constructed from reusable components (RCs), from which additional meta-attributes were derived (algorithm descriptions). Also, as a result of clustering algorithm on specific dataset internal evaluation measures (additional meta-attributes) are calculated and saved as meta-attributes. Central cloud is the most important part of metalearning system. It is responsible for ranking and selection of algorithms. Inputs in this cloud are task *x*, algorithm *a*, and performance metric *π*. Meta-attributes created for each task *x* are input for ranking and selection, as they are a basis for learning on metalevel. For most tasks performance of meta-learning system is calculated earlier, as output label, and it is available on metalevel.

### 3.2. Initial Evaluation of Component Based Metalearning System for Clustering Biomedical Data

In this section we will describe the data and the procedure for initial evaluation of the proposed system. 30 datasets gathered from original metalearning system [46] were used (http://bioinformatics.rutgers.edu/Static/Supplements/CompCancer/datasets.htm).

For the construction of the metaexamples a set of 13 meta-attributes for dataset description proposed by Nascimiento et al. [46] are used (detailed description of datasets and metafeatures can be found in Nascimiento et al. [46]).

Additionally, meta-attribute space is extended with descriptions of algorithms (four components of algorithm and normalization type described in Table 1) and internal cluster evaluation measures including compactness, global silhouette index, AIC, BIC, XB-index, and connectivity. These three types of meta-attributes (dataset descriptions, reusable components, and internal evaluation measures) form the space of 24 meta-attributes.

Component based clustering algorithms were used to define algorithm space. For that purpose 504 RC-based cluster algorithms were designed for experimental evaluation. These algorithms were built by combining already described RCs (Table 1) with 4 different normalization techniques, which lead to total of 2016 clustering experiments.

For validation of clustering models AMI (adjusted mutual information) index was used since it is recently recommended as a “general purpose” measure for clustering validation, comparison, and algorithm design [47], after exhaustive comparison between a number of information theoretic and pair counting measures. Even more, this measure is thoroughly evaluated on gene expression microarray data.

After validation of component based clustering algorithms on 30 datasets, 55326 valid results were gathered, which represent metaexample repository. Next step was identification of the best algorithm for ranking and selection of algorithms for clustering gene expression microarray data.

A procedure for ranking and selection of the best clustering algorithms is based on regression algorithms, called meta-algorithms, which predict (regression task) AMI values based on a dataset metafeatures, algorithm components, and internal evaluation measures.

In this research five meta-algorithms were used. Those are radial basis function network (RBFN), linear regression (LR), least median square regression (LMSR), neural network (NN), and support vector machine (SVM).

Estimate of quality of regression algorithms is done using mean absolute error (MAE) and root mean squared error (RMSE). Validation of results is done using 70% of dataset for training the model and the remaining 30% for testing.

Performance of each algorithm, in terms of MAE and RMSE, and best values are shown in bold (Table 2).

**Figure 3:**
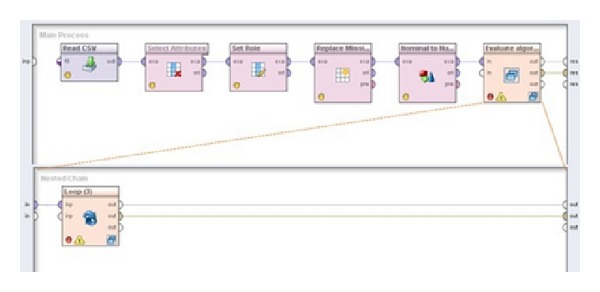
Main process for finding the best model for prediction of AMI.

Although all five algorithms showed good results, SVM, as in Table 2, gave the best performance, and this model should be used for prediction of algorithm performances for new datasets. RMSE of 0.05 and MAE of 0.034, with the smallest variance (numbers in brackets), indicate that this metamodel is applicable to the new problems since AMI measure takes values from 0 to 1 where 1 is the best. Note that with an extension of algorithm space and problem space these results could be changed and so continuous evaluation of available meta-algorithms should be done. Because of this it is important to have adequate computing infrastructure for processing big data and updating the models. Process for creating and updating the models is presented in Figure 3. Creation of model contains several steps. First, microarray metaexamples are loaded, from which only important variables are selected. After that, data preparation phase was conducted where only those attributes that are important for model building were selected, label attribute was set on AMI attribute, missing values were replaced with average value, and nominal values were transformed to numerical values using dummy coding. Modeling phase is conducted using 10-fold cross validation where the above-mentioned five algorithms were used. Every trained model is saved on hard disk, which allows its reusability.

**Figure 4:**
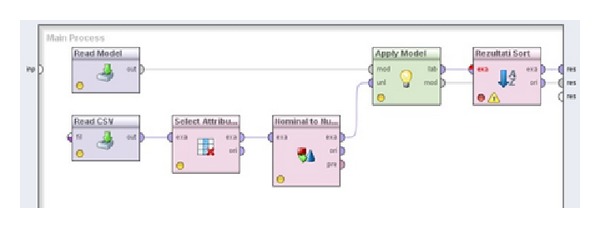
Stream for application of metalearning system on new cases.

Automatic application of the selected (in this case SVM) model is presented in Figure 4. After every update of the model or after inserting new dataset, this process needs to be updated. Saved model, in this case SVM, is loaded and applied on new dataset. Results gathered are sorted and exported. Detailed information can be found in Radovanovic et al. [48].

## 4. Extended Cloud Based Model for Big Data Analysis

Dai et al. [16] addressed the problems of storing and analysis of biomedical data by proposing a cloud based model for analysis of biomedical data and it is composed of four service categories:

(i) data as a service (DaaS),
(ii) software as a service (SaaS),
(iii) platform as a service (PaaS),
(iv) infrastructure as a service (IaaS).

DaaS is group of cloud services which enables on demand data access and provides up-to-date data that are accessible by a wide range of devices that are connected over the web. In case of bioinformatics, Amazon Web Services (AWS) provides repository of public archives (data sets), including GenBank, Unigene, Ensembl, and Influenza Virus, which can be accessed from cloud based applications.

Since bioinformatics requires a large diversity of software tools for data analyses, the task of SaaS in bioinformatics is to deliver and enable remote access to software services online. Thus, installation of software tools on desktop computer is no longer required. Another one advantage of using SaaS is enabling much easier collaboration between dispersed groups of users.

Platform as a service (PaaS) should offer a programmable environment for users in order to develop, test, and deploy cloud applications. Computer resources scale automatically without user interference. In most PaaS services, besides programming environment, database and web server are available.

Since most medical institutions do not have computing resources, such as CPU, IaaS offers a full computer infrastructure delivering virtualized resources. Those virtualized resources can be operating systems, RAM, CPU, or other computer resource.

**Figure 5:**
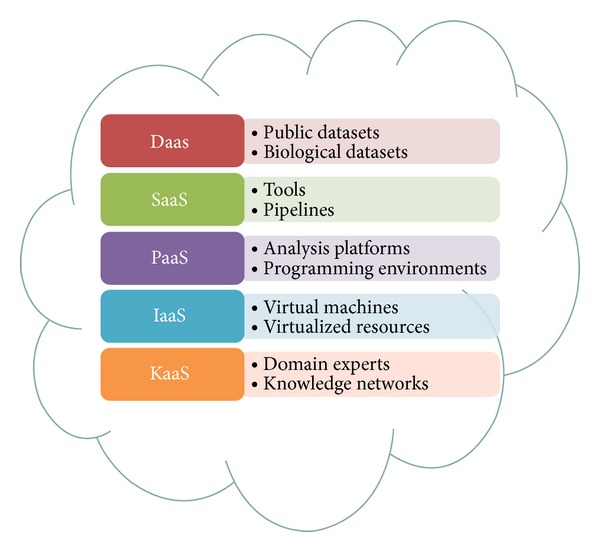
Cloud based system [16] integrated with KaaS extension [17] for analysis of biomedical data.

Lai et al. [17] introduced a new service model in cloud computing—the knowledge as a service (KaaS) that facilitates the interoperations among members in a knowledge network. Extended model is depicted on Figure 5.

**Figure 6:**
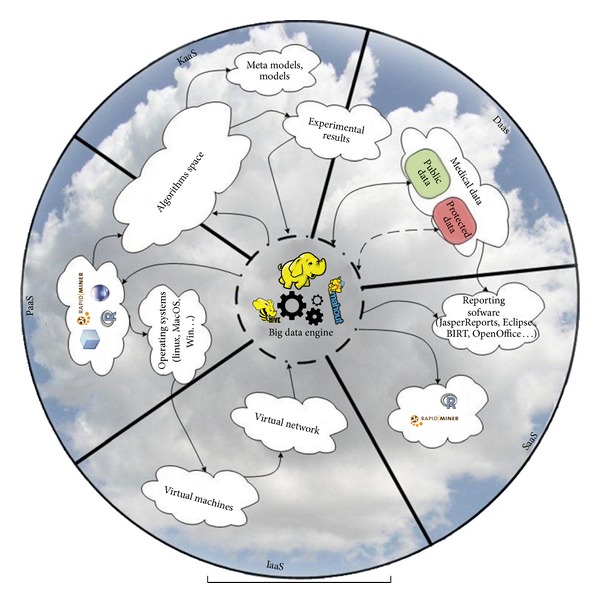
Cloud based system for predictive modeling of biomedical data.

This new service model relies on data created in a collaboration process of domain experts. It was recognized as a new form of cloud service and categorized as KaaS. In a nutshell, such approach is data driven and lacks higher order structure in order to provide knowledge as a service. This implies that KaaS in this form is provided by human, domain experts. We extend this class of services with data and model approach. By combining data driven models and expert knowledge that is stored in unstructured data like documents, notes, and collaborations, knowledge is created and offered to cloud users. Specifically, we extend models of [16, 17] by including big data technologies, platforms for data mining, and metamodels for ranking and selection of the best algorithms for biomedical data mining. System components are displayed on a diagram (Figure 6) and classified according to service type directed towards end user. Big data engine provides data storage and access and it is a basis of each class of services (diagram center).

Key component of KaaS is metalearning algorithms and selected algorithms for clustering biomedical data. Both are represented by algorithm space component, and both are accompanied by their describing metamodels. These metamodels are used in runtime for ranking and selection of best algorithms for the new problem (dataset). Experimental results provide knowledge on performances of each algorithm with meta-attributes on each dataset. Data flows are kept using big data engine such as Hadoop. Segment of experimental results component lies on DaaS since these meta-attributes can be provided as a data service. DaaS provides biomedical data. It is divided into protected and public segments.

Cloud approach provides data accumulation and higher availability of data to interested parties (with rights of access implied). Interested parties can be found among not only medical employees and researchers but also community using different software tools to access data using DaaS.

Central part of the circle (Figure 6) contains components of a big data engine (HDFS, Hive, and Mahout) where all the data are centralized (medical data, metadata, and algorithms performances). Additionally, big data engine provides interfaces for data manipulation and analysis. Apache Hive [49] is data warehouse software built on top of Hadoop, used for querying and managing large datasets residing in distributed storage. Apache Mahout [50] is also built on top of Hadoop and is used as a scalable machine learning library for classification, clustering, recommender systems, and dimension reduction.

Our solution provides software for data analysis in a service form (SaaS) with respect of SOA dependability [51]. Additionally, third party software can easily become an integrated part of SaaS (RapidMiner [52], R [53], or others).

These software solutions are recommended because they have a direct interface for access and analysis of big data (e.g., Radoop [54] allows using visual RapidMiner interface and has operators that run distributed algorithms based on Hadoop, Hive [49], and Mahout [50] without writing any code).

Most medical institutions require not only computing resources, such as CPUs, but also communication infrastructure; these components are offered as a part of IaaS, in a form of virtualized resources. Higher level of service rests on using platform such as variety of operating systems. But platform can also provide tools for development of algorithms and applications (Eclipse, Netbeans, RapidMiner, R…). The circle is closed by development of new algorithms and also for algorithm deployment and execution. For this reason algorithms space component is partly situated in PaaS space.

## 5. Conclusion and Future Research

In this paper we proposed a cloud based architecture for storing, analysis, and predictive modeling of biomedical big data. Existing service based cloud architecture is extended by including metalearning system as a data and model driven knowledge service. As a part of the proposed architecture, we provided a support for community based gathering of data and algorithms that is an important precondition for quality of metalearning. Advancement of this research area and adding new value are enabled through platform for development and execution of distributed data mining processes and algorithms. Finally, we provided data and model driven decision support on selecting best algorithms for working with biomedical data.

Retrospectively, proposed solution focuses on a specific type of biomedical data, while other types still remain to be included and evaluated. Data security and privacy still remains a concern to be taken into a more serious account. In order to provide even further impact in research community, additional work is necessary on providing interoperability among potential open source components.

System was tested on microarray gene expression data, with specific meta-attributes for this data type (e.g., chip type). Further efforts will be made to include other types of biomedical data. This will be done by identifying specific meta-attributes that fit newly included data types. Additionally, as a further work, integration with OpenML [55] platform, used for storing and gathering datasets and clustering algorithm runs, is planned. This platform provides a base for a community to share experiments, algorithms, and data. Significant clustering algorithm meta-attributes can be extracted and used for updating our metalearning system.

## Figures and Tables

**Table 1:** Sub-problems and RCs for generic clustering algorithm design.

Sub-problem	Reusable components
Initialize representatives	DIANA, RANDOM, XMEANS, GMEANS, PCA, KMEANS++, SPSS

Measure distance	EUCLIDEAN, CITY, CORREL, COSINE

Update representatives	MEAN, MEDIAN, ONLINE

Evaluate clusters	AIC, BIC, SILHOU, COMPACT, XB, CONN

**Table 2:** Meta-algorithm performance.

Algorithm/Error	RMSE	MAE
RBFN	0.143	0.109 (±0.092)
LR	0.111	0.086 (±0.070)
LMSR	0.265	0.094 (±0.248)
NN	0.101	0.064 (±0.078)
SVM	**0.050**	**0.034 (±0.036)**
